# High-velocity laser Doppler vibrometry measurements on an aluminum nitride bimorph wedge resonator

**DOI:** 10.1038/s44172-026-00595-7

**Published:** 2026-02-05

**Authors:** Zihuan Liu, Xiaoyu Niu, Ehsan Vatankhah, Yuqi Meng, Seunghwi Kim, Ruochen Lu, Andrea Alù, Neal A. Hall

**Affiliations:** 1 Chandra Family Department of Electrical and Computer Engineering, The University of Texas at Austin, Austin, TX USA; 2 RTX BBN Technologies, Cambridge, MA USA; 3 Photonics Initiative, City University of New York, New York, NY USA; 4 Physics Program, Graduate Center, City University of New York, New York, NY USA

**Keywords:** Electrical and electronic engineering, Mechanical engineering, NEMS, Applied physics

## Abstract

Recent advances in microelectromechanical systems (MEMS) have advanced inertial sensor technology. For resonant gyroscopes, sensitivity scales with the maximum velocity of the resonating mass, as higher velocities amplify the Coriolis force for faster and more accurate inertial signal detection—critical in navigation applications. Conventional MEMS remain in linear regimes, with velocities typically below 5 m/s. A recent Defense Advanced Research Projects Agency (DARPA) initiative challenges researchers to push resonator speeds toward material fracture limits, targeting up to 200 m/s and exploring regimes dominated by strong nonlinearities. This work investigates velocity limits in piezoelectrically driven mechanical resonators imposed by nonlinear dynamics and material constraints. We experimentally demonstrate an AlN bimorph wedge resonator reaching 50 m/s, achieving a ten-fold improvement over current limits. These results highlight the feasibility of operating MEMS devices at much higher velocities, paving the way for next-generation inertial sensors with increased performance. The resonator operates at a higher-order mode near 1.81 MHz, with clear evidence of Duffing-type nonlinearities at large drive amplitudes, as confirmed in time-domain and frequency-domain measurements.

## Abstract (web summary)

Zihuan Liu and colleagues reported measurements on a MEMS resonator that reaches a mechanical velocity of 50 m/s, a ten-fold improvement over typical devices. These results show that operating MEMS near limits could enable much more sensitive inertial sensors for navigation.

## Introduction

Over the past two decades, advancements in microelectromechanical systems (MEMS) sensors have transformed inertial sensors for portable consumer electronics devices. Despite notable progress in commercial applications, critical challenges persist in achieving the performance required for high-precision, man-portable inertial sensing in GPS-denied environments. A central performance metric is the sensor sensitivity, defined as the ratio between the inertial stimulus and the generated output signal. It is well known that Coriolis-based MEMS gyroscopes require at least two degrees of freedom (DOF). The equations below were adapted from standard 2-DOF equations of motion of a vibratory rate gyroscope^1^, with added cubic nonlinearity:

1 $$m\ddot{w}+{c}_{w}\dot{w}+{k}_{w}w+{k}_{w}{w}^{3}={\tau }_{w}$$

2 $$m\ddot{u}+{c}_{u}\dot{u}+{k}_{u}u={\tau }_{u}-2m{\Omega }_{v}\dot{w}$$

Where *m* is the mass, *w* and *u* are the displacements along driving and sensing directions respectively, *c* is the damping coefficient, *k* is the combined stiffness, *τ* is the external force, and *Ω*_*v*_ is the angular velocity vector. The higher the velocity, the greater the Coriolis force, improving the sensitivity. Conventional MEMS sensors typically operate with “$\dot{w}$” in a linear regime, i.e., with small oscillation velocity “$\dot{w}$” referring to equation (1). This inherently limits the sensor’s sensitivity by resulting in a small Coriolis force.

Enhanced sensitivity is essential for applications that demand precise detection of inertial signals with high bandwidth. For example, conventional gyroscopes may require extended integration periods to resolve subtle inertial phenomena, such as the rotation of the Earth. In contrast, sensors with an increased sensitivity could reduce these integration times, enabling near-real-time measurement capabilities. Such improvements in temporal resolution are critical for dynamic applications, including unmanned aerial vehicles and handheld navigation systems, where rapid and accurate inertial information is vital for situational awareness and operational effectiveness.

The limitation of current sensor designs primarily stems from entrenched industrial practices that favor linear operation—a paradigm well-suited for the automotive sector but inadequate for the more demanding needs of advanced defense and high-performance navigation applications. Consequently, increasing the sensor sensitivity represents a crucial requirement for next-generation inertial sensing and positioning systems, with the potential to enhance performance in complex and GPS-denied operational environments.

The Defense Advanced Research Projects Agency (DARPA) program Nimble Ultrafast Microsystems (NIMBUS) is an initiative aimed at exploring the limits of achievable velocity in a MEMS resonator^2^. This initiative aims to investigate MEMS operating near their break limit to explore what resonator velocity is possible. The body of work on MEMS inertial sensors is so large that an attempt to provide a compact review in this introduction would be superficial. A recent review article covering only MEMS resonant accelerometers (not gyroscopes) cites more than 200 prior works^3^. Reporting resonator velocity has not been common, but a notable exception is Ref. ^4^, which reports a large driving velocity equal to 3.39*m*/*s*. In this work, we report a velocity of 50 m/s (100 m/s p2p) from a piezoelectrically-driven MEMS resonator, as verified with laser Doppler vibrometer (LDV) measurements. The device is a wedge-shaped aluminum nitride (AlN) bimorph resonator, approximately 1 *μ*m thick and 490 *μ*m in length. While exploring maximum velocity, we incidentally drive the device deep into a nonlinear operating regime, with Duffing nonlinearity observable in time and frequency domain data.

This work focuses on the scientific challenges to achieve, demonstrate, and experimentally characterize piezoelectric resonators driven to high velocity. Practical application towards inertial sensors requires meeting additional challenges not addressed here. These challenges include designing a structure with orthogonal sensing capability, as well as showing stable, sustained operation in an oscillator loop.

## Methods

### Device description

**Fig. 1:**
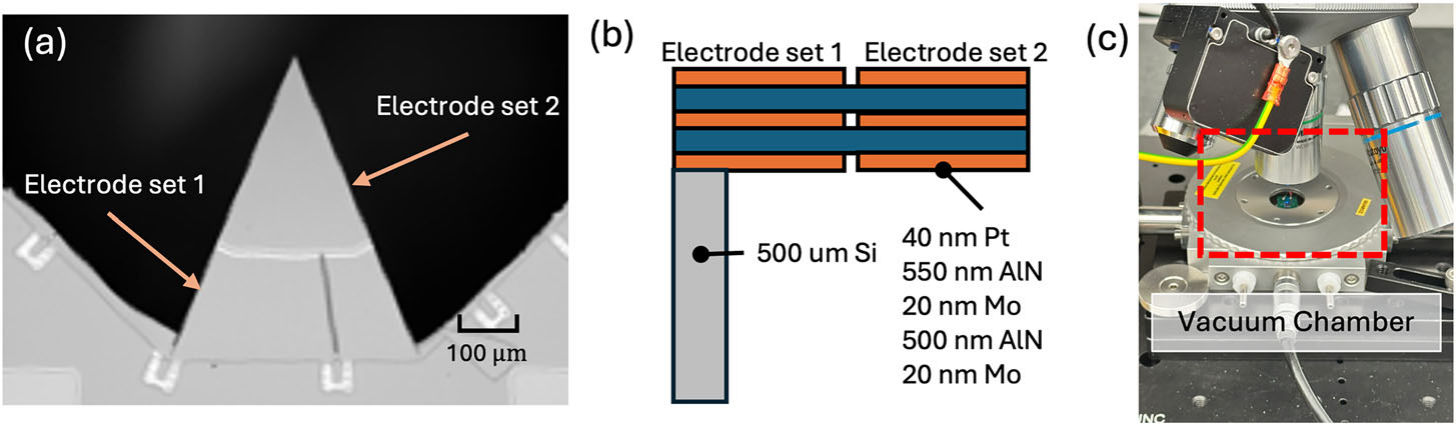
Structural design and experimental characterization setup of the piezoelectric wedge resonator. **a** Top-down optical micrograph of the resonator. **b** Cross-sectional schematic illustrating the AlN bimorph structure. The center electrode is grounded, while the top and bottom electrodes are tied to a common drive polarity. **c** Experimental setup featuring the device mounted within a low-profile Linkam vacuum chamber. Note that only electrode set 1 is driven for all measurements.

The device under test (DUT) is a wedge-shaped piezoelectric cantilever resonator fabricated on a silicon handle wafer. The resonator is approximately 490 *μ*m long and tapers in width from the anchor to the tip. The structural stack consists of an AlN bimorph with a total thickness of approximately 1 *μ*m. As illustrated in Fig. 1(b), the stack comprises three molybdenum electrode layers separated by two AlN piezoelectric layers. The center electrode is grounded to serve as a common reference, while the top and bottom electrodes are electrically tied to a common drive polarity to induce out-of-plane bending moments.

### Experimental setup

The experimental setup is depicted in Fig. 1(c). The diced resonator chip was wire-bonded to a custom printed circuit board (PCB) and mounted inside a low-profile vacuum chamber (Linkam BCS196, Linkam Scientific Instruments). The chamber was evacuated using a rotary vane pump (Edwards RV series) to a base pressure of approximately 5 mTorr to minimize air damping. Pressure levels were monitored using a Pfeiffer Vacuum Active Pirani Gauge (TPR 280) throughout the experiments.

To drive the resonator into the high-velocity nonlinear regime, the excitation signals generated by the vibrometer’s internal function generator were amplified using a high-speed, high-voltage amplifier (Electronics & Innovation A075). This amplification was necessary to achieve the drive voltages up to 360 V required for the measurements presented in Fig. 4.

### Laser Doppler vibrometry and data acquisition

Out-of-plane velocity measurements were performed using a Polytec MSA-600X scanning laser Doppler vibrometer (sLDV). The system provides a measurement bandwidth from DC to 300 MHz and a maximum velocity limit of 200 m/s. To accommodate the large tip displacements and minimize geometric optical errors caused by the high curvature of the wedge tip, measurements were conducted using long-working-distance 20× (NA = 0.42) and 50× (NA = 0.55) objectives.

**Fig. 2:**
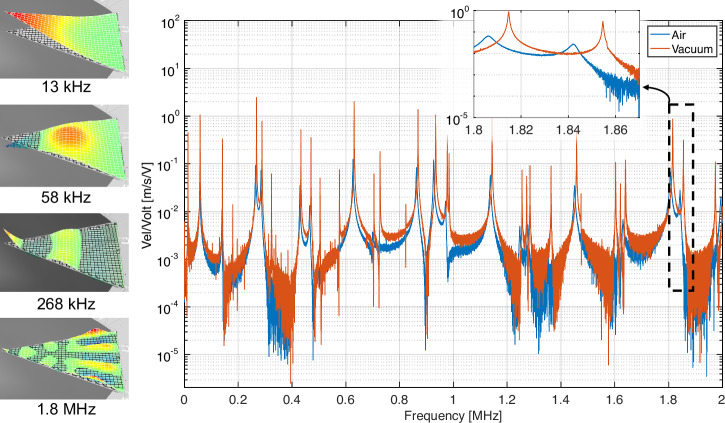
Small-signal response of the device in air and vacuum (~5 mTorr).

**Fig. 3:**
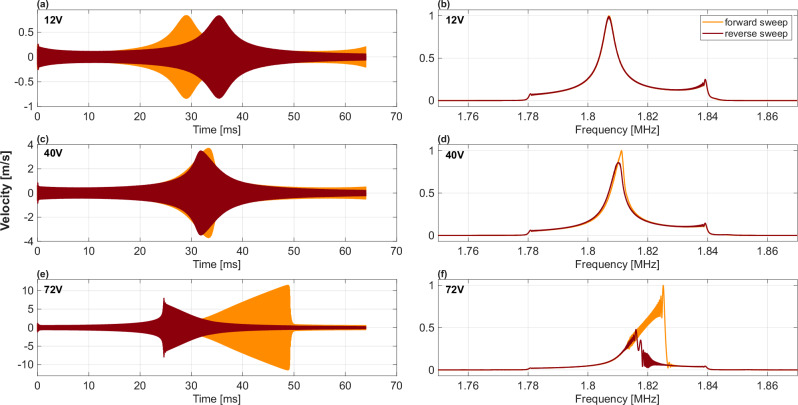
Velocity waveforms measured in air and their corresponding Fast Fourier Transforms (FFTs) magnitude spectra for constant amplitude input. The input waveform is a constant amplitude continuous frequency sweep of 64 ms duration, spanning frequencies from 1.78 MHz to 1.84 MHz. **a**, **c**, **e** Time-domain velocity waveforms were measured at drive voltages of 12 V, 40 V, and 72 V, respectively. Orange traces indicate the forward sweep, and maroon traces indicate the reverse sweep. **b**, **d**, **f** Corresponding frequency-domain FFTs of the waveforms in (**a**), (**c**), and (**e**), respectively. The device behavior is linear at low drive (12 V) and becomes increasingly nonlinear at high drive (40 V and 72 V).

**Fig. 4:**
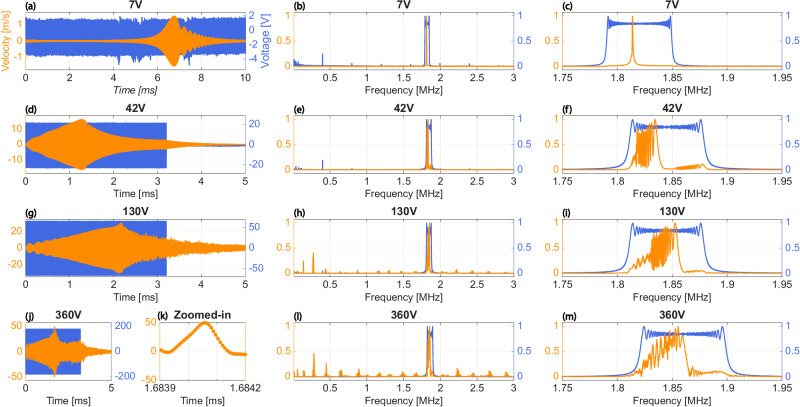
Velocity waveforms in response to constant-amplitude forward frequency-swept burst-chirp voltage inputs, measured in vacuum (~5 mTorr). **a**, **d**, **g**, **j** Time-domain measurements showing the input voltage (blue traces, right axis) and resonator tip velocity (orange traces, left axis) for drive amplitudes of 7 V, 42 V, 130 V, and 360 V, respectively. **b**, **e**, **h**, **l** Normalized Fast Fourier Transforms (FFTs) magnitude spectra of the input voltage (blue) and output velocity (orange) plotted over a wide frequency range (0–3 MHz) for the corresponding drive levels. **c**, **f**, **i**, **m** Zoomed-in frequency spectra (1.75–1.95 MHz) highlighting the resonance peak and nonlinear distortion near the fundamental mode. **k** A magnified view of the time-domain velocity peak from the 360 V measurement. **j** illustrating the adequate sampling rate for these measurements.

For the small-signal characterization (Fig. 2), a broadband periodic chirp excitation was applied, and the response was averaged over 5 chirp repititions to improve the signal-to-noise ratio. For the large-signal nonlinear characterization (Figs. 3, 4), we utilized burst-chirp waveforms to manage thermal loading and prevent dielectric breakdown. The burst signals consisted of a 10% duty cycle with a sweep duration of 64 ms. The frequency sweeps were continuous rather than discrete stepped-sine to fully capture the transient jump phenomena associated with the Duffing nonlinearity.

Data acquisition and signal processing were performed within the Polytec PSV software environment (version 10.2). The velocity frequency response was obtained by computing the Fast Fourier Transform (FFT) of the time-domain velocity signal, normalized by the FFT of the measured input voltage waveform. A rectangular window was applied to the time-domain data to minimize spectral leakage.

### Numerical simulation

To validate the experimental findings, the nonlinear dynamics were modeled using the Duffing equation. The lumped-parameter coefficients (mass *m*, stiffness *k*, and damping *λ*) were extracted from the linear regime measurements using the method described by Ayela et al. (See Supplementary Note 1). The equation of motion was solved numerically using COMSOL Multiphysics (version 6.3) via the 0D ODE interface. The time-dependent solver was configured with an implicit BDF stepping method to ensure stability during the rapid transient jump events observed in the forward and reverse sweeps.

### Use of Large Language Models

This manuscript was edited for clarity and coherence using ChatGPT-5 (OpenAI). The authors reviewed and revised the output to ensure accuracy and retain full responsibility for the content.

## Results

### Measurements

Figure 2 presents the measured small-signal frequency response of the device in air. The vertical axis is the measured velocity at the tip of the wedge, normalized to input voltage. These data are obtained by applying a broadband chirp voltage waveform to the device and measuring the resulting velocity waveform at the tip. In this article, the terms 'chirp' and 'sweep' are used interchangeably. In all experiments, sweeps are continuously rather than discretely incremented. The FFT of the velocity waveform is normalized to the FFT of the input waveform to obtain the frequency response. The sLDV automates the repetition of this measurement at predefined points across the resonator’s surface, and subsequent synthesis of all measurement points provides the vibration profile at any selected frequency. Several vibration modes are presented as inset images in Fig. 2.

Next, a narrowband excitation is applied to a single mode to explore the resonator’s achievable maximum velocity. We select 1.81 MHz resonance mode for study in this article since this mode has one of the largest peaks in the small-signal response presented in Fig. 2. In a first set of experiments, a narrowband continuous-wave sweep waveform is provided to the device. The duration of the sweep input is 64 milliseconds. Two different sweep waveforms are input; a forward sweep with frequency swept low to high, and a reverse sweep with frequency swept high to low. The magnitude of the FFT of the input waveform is the same in each case and is presented in Fig. 3(a) labeled “input.” The frequency bounds of the sweep are 1.78 MHz and 1.84 MHz, as discernible from Fig. 3(a). Velocity waveforms in response to forward and reverse sweeps are presented in Fig. 3(b), and their FFTs are depicted in Fig. 3(a). The waveforms in Fig. 3(b) are identical except for time reversal, and their respective FFTs are identical as observed in Fig. 3(a), as expected for a linear system. The resonance quality factor Q is 446.84 for this measurement in air.

The same experiment is repeated using a larger input voltage, with results presented in Fig. 3(c) and (d). The FFTs in Fig. 3(c) for forward and reverse cases are different, with the forward sweep resonance displaying asymmetry and distortion. The asymmetry of the resonance is explainable by Duffing nonlinearity and is consistent with observations of other research teams^5–8^. The input is further increased, with results presented in Fig. 3(e) and (f). Distinctly different behavior is observable in the time and frequency domains for the forward vs. reverse case, where the frequency response bends in opposite directions in either case, demonstrating the characteristic jump phenomenon of Duffing nonlinearity. A velocity amplitude greater than 10 m/s is reached in the forward sweep, as noted in Fig. 3(f). The system is highly nonlinear in this large signal regime.

Figure 4 presents the results from a second set of experiments conducted in a vacuum with a chamber pressure of approximately 5 mTorr. The left column of Fig. 4 presents a set of input waveforms with the corresponding velocity waveforms. Figure 4 also presents the FFT of the input and output waveforms using the complete frequency axis for the images in the middle column, and limited frequency for images in the right column to better show details of the input and output spectrum near the resonance. The upper and lower frequency limits used in the excitation waveforms in Fig. 4 left column are discernible from their corresponding FFTs presented in Fig. 4 right column. The frequency bounds are slightly different in each experiment by necessity in order to ensure full coverage of the resonance and Duffing nonlinearity, and in turn, to achieve a maximum velocity in observed time-domain waveforms. The input waveforms in Fig. 4(d), (g), (j) are narrowband forward burst-chirps with 10% duty cycle and with upper and lower frequency chosen to span the 1.81 *MHz* resonance. The switch to burst waveforms for these high input voltages was to limit the time-averaged electrical power input to the device. The FFT in Fig. 4(c) shows a single resonance peak with Q = 4559.86, approximately 10X higher in vacuum than in air. Such a single resonance peak is expected for a linear system. The progression of FFTs in Fig. 4(f), (i), (m) displays behavior anticipated from a system with strong Duffing nonlinearity. Further, the full spectrum visible in Fig. 4(l) displays many tones outside of the excitation band, exemplifying a highly nonlinear system. Most notable from these measurements, a velocity equal to 50 m/s is observed in Fig. 4(j).

**Fig. 5:**
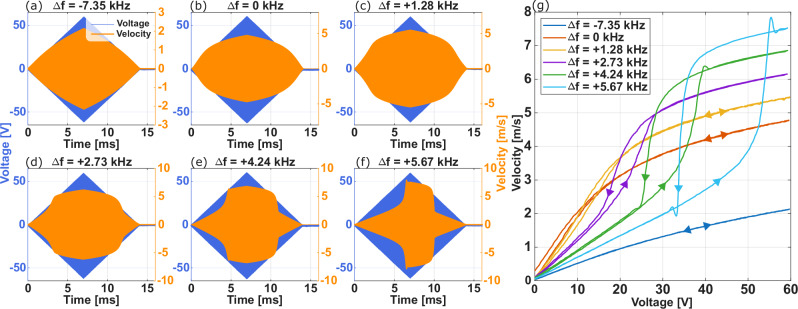
Nonlinear dynamic response and hysteresis characterization of the piezoelectric MEMS resonator. **a**–**f** show the time-domain measurements performed in air with fixed frequencies and ramped amplitude. *Δf* is the difference between the driving and linear (i.e., small-signal) resonance frequency. As *Δf* increases, the nonlinearity becomes more prominent, leading to a sharp velocity increase. **g** presents the asymmetry of the velocity surge and plunge, highlighting the emergence of hysteresis loops.

In the experiments presented thus far, the amplitude of excitation was held fixed, and the frequency was linearly varied up or down in a narrowband encompassing the resonance frequency. In the next set of measurements conducted in air, the frequency of excitation is kept fixed, and the amplitude is linearly increased and decreased. This process is repeated for several frequencies near the resonance, with the results illustrated in Fig. 5(a)–(f). The nonlinearity of the resonator becomes evident in the envelope of the tip velocity, which deviates from the linear ramp-up and ramp-down of the applied voltage. Moreover, for frequencies above the resonance, as shown in Fig. 5(e) and (f), this nonlinearity is apparent as a sharp velocity increase during the voltage ramp-up phase and an abrupt velocity decrease during the ramp-down phase. Notably, these two sudden changes in velocity exhibit asymmetry. To more clearly demonstrate the nonlinear jump phenomenon in velocity, the envelope velocities from Fig. 5(a)–(f) are plotted against the input voltage envelope in Fig. 5(g). The asymmetry of the velocity surge and plunge, and consequently the formation of a hysteresis loop, is distinctly visible in Fig. 5(g), where arrows indicate the direction of increasing and decreasing applied voltage.

### Simulation

The nonlinear behavior observed in these measurements can be effectively modeled using a forced, damped harmonic oscillator with cubic nonlinearity, commonly known as the Duffing equation.^9^^,^^10^^,^^11^ The cubic restoring force in the Duffing model can explain nonlinear behaviors such as spring softening and stiffening, and thus leading to the jumping phenomenon and the bending of the frequency response curve as has been observed in our measurements^12^. In many bending mode resonators, the cubic Duffing stiffness term is primarily a geometric nonlinearity that arises from mid-plane stretching of the diaphragm at large transverse deflection or rotation, which results in a hardening nonlinear response.^13^^,^^14^^,^^15^^,^^16^ This system is governed by the second-order differential equation having the form

3 $$\ddot{w}+\lambda \dot{w}+{\omega }_{0}^{2}w+\beta {w}^{3}=\frac{{F}_{in}(t)}{m}$$

Where *λ*, *ω*_0_, *β*, *w*, *m*, *and* *F*_*in*_ are the damping coefficient, the resonance angular frequency, nonlinear stiffness coefficient, the displacement, mass and external driving force, respectively.

To model our measurements with the Duffing equation, we first focused on estimating key parameter values, such as the nonlinear stiffness coefficient, using the data illustrated in Fig. 3. To achieve this, we applied an approximation model adapted from Ayela et al.^17^ to directly derive these parameters from the linear and frequency-shifted velocity responses depicted in Fig. 3(a) and (e). The velocity frequency response was then converted to displacement, enabling seamless application of the Ayela et al method. The resulting simulation parameters are summarized in Table 1, with a comprehensive derivation provided in Supplementary Note 1.

**Table 1:** Simulation parameters

*λ*	1.549 × 10^4^s^−1^
*β*	3.613 × 10^24^m^−2^s^−2^
*f*_0_ = *ω*_0_/(2*π*)	1.807 × 10^6^Hz
*a*_*in*_ = *F*_*in*_/*m*	2.685 × 10^5^m/s^2^

Once the parameters were established, we used the Mathematics interface in COMSOL Multiphysics to numerically solve the nonlinear Duffing equation. The driving stimulus was configured as a chirp signal, precisely matching the one employed in our experimental measurements.

**Fig. 6:**
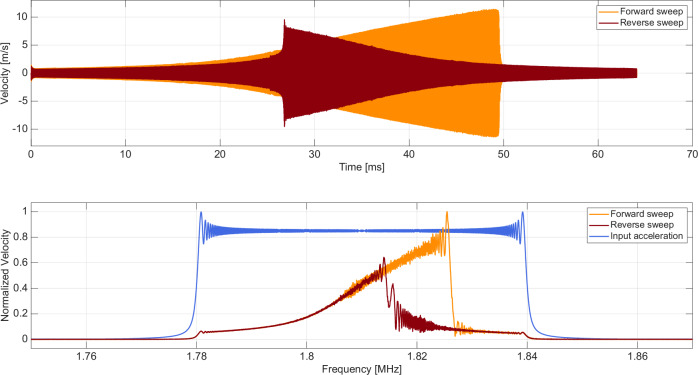
Simulation of the device tip velocity in response to constant-amplitude forward and reverse frequency sweeps under large drive input. This simulation matches the corresponding data presented in Fig. 3(e) and (f).

The normalized time-domain and frequency-response curves from our simulations are presented in Fig. 6 and align closely with the experimental data.

## Discussion

Several devices were tested, and the average electrical breakdown occurs near 400 V for the 10% duty-cycle burst-chirp waveforms summarized in Fig. 4. The corresponding E field is 400 *V*/1 *μm* = 4 × 10^8^V/m. This value is of the same order as published breakdown voltages for AlN films, but a factor of 2X to 3X higher which may be attributed to test conditions unique to these experiments (frequency and duty cycle). We therefore believe that, considering breakdown voltage limitations, 50 m/s is the approximate maximum velocity achievable for this wedge resonator as tested under the described conditions and using the selected mode at 1.81 MHz. It is interesting to explore how close the DUT was to mechanical failure. To explore this, we estimate the maximum strain experienced by the resonator during the 50 m/s tip velocity measurement. Strain at the top surface of a bending structure is related to out-of-plane deflection *u* as:

4 $$\varepsilon =-\frac{h}{2}\frac{{d}^{2}u}{d{x}^{2}}$$

**Fig. 7:**
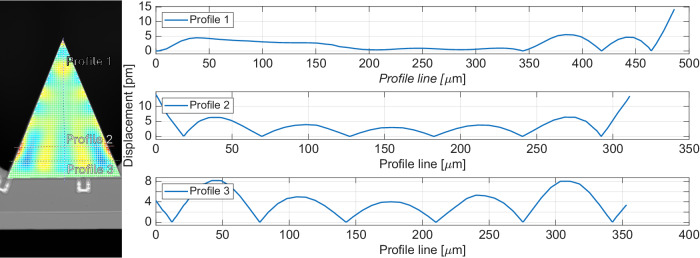
Measured deflection profiles and computed strain along 3 profiles.

The small-signal LDV scan at the 1.81 MHz mode enables the computation of u along any contour. Figure 7 presents the measured deflection along 3 different profiles using the small-signal drive measurement when the tip velocity is 1.5 mm/s. Strain scales with displacement and displacement scales with velocity at a particular frequency, per *v* = *jωu*. Measuring the displacement profile at 50 m/s would be more direct, and would circumvent needing to scale small-signal results to estimate the strain. However, a high magnification lens was needed for the large signal measurements due to extreme tip angle, and this lens has a limited field of view, preventing a scan of the entire resonator surface. The maximum strain calculated based on the displacement plots in Fig. 7 is approximately 1 × 10^−7^. The ratio of max strain to tip velocity is *R* = 1 × 10^−7^/1.5 mm/s. This ratio R is multiplied by 50 m/s tip velocity to yield a strain equal to 0.0033. This strain, which we believe to be the maximum strain experienced at 50 m/s, is approximately 1/2 the published break strain for AlN^18^. From this, we conclude that the beam was within 50% of mechanical failure during the measurement in Fig. 4(j).

**Fig. 8:**
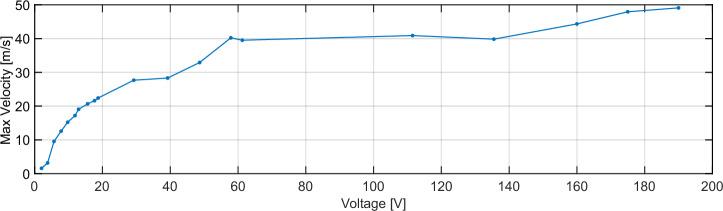
Measured maximum tip velocity vs input voltage in vacuum (~5 mTorr), obtained using constant-amplitude, forward-swept burst chirp voltage inputs described in Fig. 4.

Figure 8 presents the measured tip velocity vs. input voltage, using forward-swept burst-chirp inputs described in Fig. 4. The curve shows a linear relationship for small-signal inputs, followed by a significant departure from linearity for velocities above 15 m/s. We also wish to note the challenges experienced thus far in making high-velocity measurements with LDV. The large curvature of the vibration profile near the wedge tip can deflect the reflected laser light out of the objective’s aperture or capture region. When this happens, time-domain waveforms show individual lone data points/sample values that fall outside of established trends with erroneously high velocity. The data presented in this article were closely inspected to ensure no such data points.

## Summary

This study experimentally demonstrates achieving cantilever tip velocities exceeding 50 m/s in a MEMS resonator. Such extreme tip velocities produced large tip angles, requiring specially designed testing environments with a large objective for observation. These results are enabled by careful consideration of nonlinear resonator dynamics, including the drive-amplitude dependence of resonance frequency and the impact of sweep direction on system response trajectory. The use of swept waveforms was essential to probe the device’s nonlinear response and capture the complex dynamics associated with increasing drive amplitude. Figure 5 illustrates the distinct nonlinear behaviors as the frequency detuning from the small-signal resonance is varied. At low detuning, velocity saturation is observed, while increasing detuning leads to pronounced jump phenomena and hysteresis loops typical of nonlinear oscillators. The asymmetric surge and plunge of velocity, clearly resolved in Fig. 5, not only affirm the predicted nonlinear oscillator models but also provide crucial insight for maximizing output velocity in MEMS resonators. This work bridges MEMS experimental observations nonlinear structural mechanics theory, offering demonstrations of asymmetric velocity hysteresis loops in this context.

The reported on-chip resonator velocity of 50 m/s exceeds previously published values. At this velocity, the resonator is driven at 90% of the electrical breakdown limit and within 50% of the predicted mechanical failure threshold. We therefore conclude that 50 m/s is close to the maximum achievable velocity for this resonator in the chosen mode. Significant curvature at the resonator tip introduces geometric-optics challenges for LDV measurements. The device used for the presented study was not specifically designed for high velocity. Rather, the device was repurposed from a different application. That said, a few features motivated our interest in investigating this particular resonator. The resonator is entirely composed of active (i.e., piezoelectric) material. Further, the resonator has a tapered beam width, and we anticipated high tip velocity considering continuity of momentum along the beam length. The results demonstrate that the device effectively illustrates several nonlinear oscillator dynamics, including amplitude- and frequency-ramp hysteresis. Future work will explore additional modes of this device, evaluate alternative devices, and develop engineered solutions to the measurement challenges identified in this work.

## Supplementary information

Supplementary information

## Data Availability

The data that support the findings of this study are available from the corresponding author upon reasonable request.
