# Supplementary material for: High-velocity laser Doppler vibrometry measurements on an aluminum nitride bimorph wedge resonator
Source: Commun Eng. 2026 Feb 5;5:48. doi: 10.1038/s44172-026-00595-7 (PMC12982748; doi:10.1038/s44172-026-00595-7)
Supplement: Supplementary file 1 — Supplementary information [file 44172_2026_595_MOESM1_ESM.pdf]

Supplementary Note 1: Derivation of simulation parameters

The figure below, adapted from Reference 13 of the original manuscript, shows the relationship between amplitude and frequency shift for both a damped harmonic oscillator (thin solid line) and a Duffing anharmonic oscillator (thick solid line). The said reference outlines a method for approximating the parameters in the anharmonic oscillator equation. For a comprehensive derivation of the plot and the underlying parameterization, the reference could be consulted.

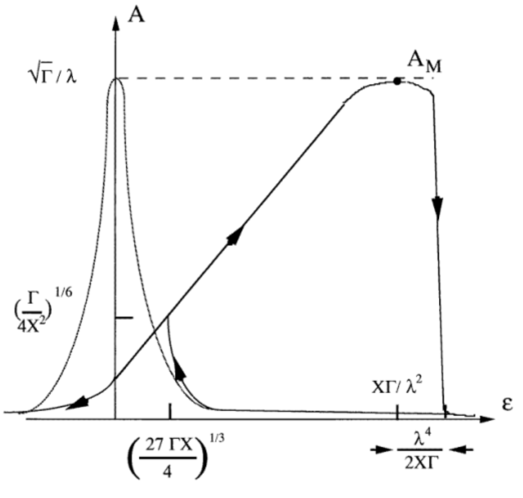

The x axis ε is the difference between the current's frequency ω and the main frequency ω_o of an equivalent harmonic system, i.e., $\varepsilon = \omega - \omega_o$.

Following formulae and table are borrowed from [1] which provides Ayela's method in a more readily computable presentation. Important parameters in the figure are summarized in the following table:

	**Amplitude [mV]**	**Frequency [Hz]**
**Forward sweep**	$A_F = \frac{\sqrt{\Gamma}}{\lambda}$	$\Delta f_F = \frac{1}{2\pi}\left(\frac{X\Gamma}{\lambda^2}\right)$
**Reverse sweep**	$A_R = \left(\frac{\Gamma}{4X^2}\right)^{\frac{1}{6}}$	$\Delta f_R = \frac{1}{2\pi}\left(\frac{27X\Gamma}{4}\right)^{\frac{1}{3}}$

Using the relations for forward and reverse frequency shift, the damping coefficient could be estimated as follows:

$$\lambda = \sqrt{\frac{2(2\pi\Delta f_R)^3}{27\pi\Delta f_F}}$$

Furthermore,

$$\Gamma = (\lambda A_F)^2$$

$$X = \sqrt{\frac{\Gamma}{4{A_R}^6}}$$

Finally,

$$\beta = \frac{16\pi}{3} X f_o$$

Moreover, the explicit value of the mass is not essential in this context, since the governing input is acceleration (force divided by mass). The mass term is included only to clarify the origin of the corresponding term in the non-homogeneous partial differential equation.

In PMUTs the cubic Duffing stiffness term is primarily a geometric nonlinearity that arises from mid-plane stretching of the diaphragm at large transverse deflection or rotation which results in a hardening nonlinear response [2-4].

Addition of other nonlinear quadratic terms such as x^2, $x\dot{x}$, and $\dot{x}^2$ which could naturally appear in realistic MEMS and NEMS scenarios, due to actuation, detection mechanism or clamping of the transducer, does not change the frequency response curve expected from damped Duffing resonator in any perceptible way. Additional quadratic and cubic nonlinearities "merely conspire to renormalize the effective values of the coefficients used in the original equation of motion" as explained by Ron Lifshitz and M. C. Cross in [5] pp 15-17. The said treatise presents the detailed treatment of the problem. The argument could be summarized as follows:

The normalized Duffing equation given by the form

$$\ddot{x} + \epsilon\dot{x} + x + x^3 + \eta x^2\dot{x} = \epsilon^{\frac{3}{2}} g \cos(1 + \epsilon\Omega)t,$$

is surmised to have a solution of the form

$$x(t) = \frac{\sqrt{\epsilon}}{2}\left(A(T)e^{it} + c.c.\right) + \epsilon^{\frac{3}{2}} x_1(t) + \cdots,$$

following secular perturbation theory, where $c.c.$ denotes complex conjugate and $A(T)$ is the slowly varying temporal envelope. It could be shown that the equation determining the amplitude $A(T)$ is given by

$$\frac{dA}{dt} = -\frac{1}{2}A + i\frac{3}{8}|A|^2A - \frac{\eta}{8}|A|^2A - i\frac{g}{2}e^{i\Omega T}.$$

If we consider an equation of motion with all the additional nonlinearities:

$$\ddot{x} + \epsilon\dot{x} + x + \beta x^2 + \mu x\dot{x} + \rho\dot{x}^2 + x^3 + \eta x^2\dot{x} + \upsilon x\dot{x}^2 + \zeta\dot{x}^3 = \epsilon^{\frac{3}{2}} g\cos(1 + \epsilon\Omega)t,$$

After lengthy mathematical manipulations, it could be shown that the governing equation for the amplitude $A(T)$ is

$$\frac{dA}{dt} = -\frac{1}{2}A + i\frac{3}{8}\alpha_{eff}|A|^2A - \frac{1}{8}\eta_{eff}|A|^2A - i\frac{g}{2}e^{i\Omega T}.$$

which is identical to the one shown earlier where α_{eff} and η_{eff} could be tuned to meet the nonlinearities arisen in particular experimental realizations.

References

[1] Xia, F., Peng, Y., Yue, W., Luo, M., Teng, M., Chen, C.M., Pala, S., Yu, X., Ma, Y., Acharya, M. and Arakawa, R., 2024. High sound pressure piezoelectric micromachined ultrasonic transducers using sputtered potassium sodium niobate. Microsystems & Nanoengineering, 10(1), p.205.

[2] Karličić, D., Chatterjee, T., Cajić, M. and Adhikari, S., 2020. Parametrically amplified Mathieu-Duffing nonlinear energy harvesters. Journal of Sound and Vibration, 488, p.115677.

[3] Arora, N., Singh, P., Kumar, R., Pratap, R. and Naik, A., 2024. Mixed nonlinear response and transition of nonlinearity in a piezoelectric membrane. ACS Applied Electronic Materials, 6(1), pp.155-162.

[4] Xu, P. and Wellens, P., 2021. Effects of static loads on the nonlinear vibration of circular plates. Journal of Sound and Vibration, 504, p.116111.

[5] Lifshitz, R. and Cross, M.C., 2008. Nonlinear dynamics of nanomechanical and micromechanical resonators. Reviews of nonlinear dynamics and complexity, 1(1).
